# Motion Tree Delineates Hierarchical Structure of Protein Dynamics Observed in Molecular Dynamics Simulation

**DOI:** 10.1371/journal.pone.0131583

**Published:** 2015-07-06

**Authors:** Kei Moritsugu, Ryotaro Koike, Kouki Yamada, Hiroaki Kato, Akinori Kidera

**Affiliations:** 1 Graduate School of Medical Life Science, Yokohama City University, 1-7-29 Suehiro-cho, Tsurumi-ku, Yokohama, Japan; 2 Graduate School of Information Science, Nagoya University, Furo-cho, Chikusa-ku, Nagoya, Japan; 3 Department of Structural Biology, Graduate School of Pharmaceutical Sciences, Kyoto University, 46–29 Shimoadachi-cho, Sakyo-ku, Kyoto, Japan; 4 RIKEN Harima Institute at SPring-8, 1-1-1 Kouto, Sayo-cho, Sayo-gun, Hyogo, Japan; Wake Forest University, UNITED STATES

## Abstract

Molecular dynamics (MD) simulations of proteins provide important information to understand their functional mechanisms, which are, however, likely to be hidden behind their complicated motions with a wide range of spatial and temporal scales. A straightforward and intuitive analysis of protein dynamics observed in MD simulation trajectories is therefore of growing significance with the large increase in both the simulation time and system size. In this study, we propose a novel description of protein motions based on the hierarchical clustering of fluctuations in the inter-atomic distances calculated from an MD trajectory, which constructs a single tree diagram, named a *“Motion Tree”*, to determine a set of rigid-domain pairs hierarchically along with associated inter-domain fluctuations. The method was first applied to the MD trajectory of substrate-free adenylate kinase to clarify the usefulness of the Motion Tree, which illustrated a clear-cut dynamics picture of the inter-domain motions involving the ATP/AMP lid and the core domain together with the associated amplitudes and correlations. The comparison of two Motion Trees calculated from MD simulations of ligand-free and -bound glutamine binding proteins clarified changes in inherent dynamics upon ligand binding appeared in both large domains and a small loop that stabilized ligand molecule. Another application to a huge protein, a multidrug ATP binding cassette (ABC) transporter, captured significant increases of fluctuations upon binding a drug molecule observed in both large scale inter-subunit motions and a motion localized at a transmembrane helix, which may be a trigger to the subsequent structural change from inward-open to outward-open states to transport the drug molecule. These applications demonstrated the capabilities of Motion Trees to provide an at-a-glance view of various sizes of functional motions inherent in the complicated MD trajectory.

## Introduction

The complexity of protein dynamics observed in molecular dynamics (MD) simulations originates from motions associated with a wide range of sizes, amplitudes, and timescales. The difficulty in analysis of MD simulation trajectories lies in the fact that functional motions of our interest are not restricted to large collective motions but possibly small localized vibrations that are likely to be obscured by the large fluctuation of such collective motions.

To analyze the complicated dynamics, protein three dimensional structure is usually decomposed into a set of building blocks, which allows separation of timescales into slow collective motions between the blocks and fast local motions within each block. There have been proposed various methods for the determination of such building blocks based on protein dynamics [1–10], which may be categorized into the following two classes: The first is principal component analysis (PCA) and its variants [1–5], which define building blocks in terms of a few dominant eigenmodes. These methods mainly treat collective and large-amplitude motions to find dynamic domains, but are not necessarily sensitive to detect localized motions. The other class adopts clustering in terms of correlation/covariance matrix to separate the whole protein molecule into a set of highly-correlated parts in motion [6–10]. Clustering is carried out efficiently in a non-hierarchical manner, but in itself does not provide detailed information on protein dynamics such as amplitudes and correlations in motion. We need a more straightforward analysis method of MD trajectories to treat all spectra of motions including both large and local motions as well as to give an at-a-glance picture of protein dynamics which thoroughly illustrates the amplitudes and correlations in the motions of the building blocks. This kind of method would be more important with increasing system size and simulation time of target proteins or protein complexes.

Koike *et al*. developed a method of comprehensively and intuitively describing all spectra of protein motions observed between two distinct crystal structures in a protein by employing a hierarchical clustering approach [11]. This method constructs a dendrogram or a tree diagram named a “*Motion Tree*”, which illustrates hierarchically all possible rigid-body-like motions with their amplitudes, by adopting an optimally tuned linkage-rule. The Motion Tree for a pair of structures was calculated according to the distance difference matrix for clustering, **D** ^pair^ (= $\left\{D_{mn}^{pair}\right\}$), given for protein structures 1 and 2 as:

$$D_{mn}^{pair}=\left|d_{mn,1}-d_{mn,2}\right|,$$ (1)

where *d*_*mn*,1_ and *d*_*mn*,2_ are the distances between atoms *m* and *n* of protein structures 1 and 2. In the present study, we aim at extending the concept of the Motion Tree to analyze the structural ensemble generated by MD simulations. To do this, as a natural extension, the difference in distance between the two structures in Eq 1 is replaced by the standard deviation in distance fluctuations in the structural ensemble, i.e., the metric matrix for clustering, **D** (= {*D*_*mn*_}), is redefined by

$$D_{mn}={\langle \Delta d_{mn}^{2}\rangle }^{1/2},$$ (2)

where the variance in the atom pair distance, $\langle \Delta d_{nm}^{2}\rangle$, is directly calculated from the MD trajectory. Matrix **D** is then subject to hierarchical clustering to calculate the Motion Tree of the structural ensemble, in which a larger/smaller subtree represents a larger/smaller rigid body, and a node placed near the root/leaves of the tree respectively depicts larger/smaller distance fluctuations. The hierarchical description indicates that the fluctuation between two rigid bodies determined at a node illustrates the largest internal motion occurring within a domain, including the two rigid bodies, which is determined at the ancestral node. The use of a distance metric for clustering is advantageous over conventional MD trajectory analysis using PCA and calculation of the root-mean-square deviation (RMSD) because calculating inter-atomic distance is more straightforward without any need for structural-fitting procedures. Note that the same metric as Eq 2 has already been used for clustering in the previous studies [7–10]. However, their methods using non-hierarchical clustering identify a set of rigid bodies on a “single scale” using a single threshold value, while the hierarchical description in the present method enables the “multi-scale” description of protein motions illustrating the relationship between the rigid-body-like motions allocated at each node.

Here, we present three illustrative applications of the Motion Trees derived from respective MD simulations. The first is ligand-free adenylate kinase (ADK) to demonstrate how to interpret the derived Motion Tree from a dynamics point of view. In the next application, Motion Trees were calculated from two MD simulations for the ligand-free and ligand-bound forms of glutamine binding protein (GBP). GBP is a periplasmic binding protein related to the membrane transport of glutamine. We found that GBP changed its dynamics upon glutamine binding, in both large collective and small loop motions. Finally, the Motion Tree describes complicated dynamics in a huge protein, a multidrug ATP binding cassette (ABC) transporter, *Cyanidioschyzon merolae* ABCB1 (CmABCB1; a half-sized ABC transporter adopting a homodimeric architecture), in the lipid bilayer. Comparison of the Motion Trees with and without a bound drug molecule suggested the possibility that drug binding was required to trigger an onset of large scale motion from inward-open to outward-open structures.

## Methods

### Construction of Motion Tree

The Motion Tree for a structural ensemble was calculated by hierarchical clustering based on a dissimilarity matrix of variance in distance between a pair of protein atoms (Eq 2). Here, only C_α_ atoms were considered for the degrees of freedom in **D** to describe residue-level structural dynamics, even though the present method allows an arbitrary choice of atom selection to be analyzed. Except for metric matrix **D**, the details on the method to construct the Motion Tree were the same as those for the two structures described in our previous paper [11]. Here is given a brief summary.

Clusters were constructed in a bottom-up manner, i.e., starting at each residue of the protein as each cluster at the bottom. Most similar clusters with the smallest *D*_*mn*_ were selected and merged to generate a new cluster if the spatial-proximity condition, the average distance <*d*_*nm*_> < 7 Å, was satisfied. Otherwise, the next most similar clusters were considered. The dissimilarity measure was updated as follows for newly merged clusters: The dissimilarity measure for two clusters to be merged, C_1_ and C_2_, i.e., *D*_C1C2_, was defined as the average of the 20 (or the number of residue pairs when it was less than 20) largest values of *D*_*mn*_ out of all residue pairs, one from C_1_ and the other from C_2_. This linkage rule was thus optimized as an intermediate between complete and average linkages [11]. Clusters were successively constructed until they were joined into one cluster at the root.

### MD simulations

Motion Trees were constructed as described above from the MD trajectories of the three proteins in explicit solvent, ADK, GBP, and the ABC transporter. The 50-ns MD trajectories of the production runs, with the coordinate frames taken every 1 ps, were used for calculating Eq 2. The simulation models and protocols are summarized below.

#### ADK

The crystal structure of the ligand-free form of adenylate kinase (PDB: 4ake_A) [12] was used for the starting structure. The AMBER ff03 force field [13] was used as the all-atom potential energy function. A rectangular simulation box was constructed with a margin of 12 Å to the boundary of the simulation box and filled with 11,599 TIP3P water molecules [14]. The solution system contained 37,488 atoms together with four sodium ions to neutralize the simulation system. MD simulation was performed using the PMEMD module (the particle mesh Ewald method [15] for the electrostatic interactions) of AMBER [16] under constant temperature and pressure (NPT) conditions at *T* = 300 K and *P* = 1 atm using Berendsen’s thermostat and barostat [17] with a relaxation time of 1 ps. A cut-off length of 8 Å was used for the Lennard–Jones potential. The simulation time step was 2 fs with constraining bonds involving hydrogen atoms via the SHAKE algorithm [18].

#### GBP

The MD simulations of glutamine binding protein with and without a bound glutamine were performed to reveal the ligand binding contribution to GBP dynamics. The starting structures of glutamine-free and-bound GBPs were respectively taken from the crystal structures in the PDB entries, 1wdn_A and 1ggg_A [19]. The all-atom potential energy function of the AMBER ff99SBildn force field [20] was used. Rectangular simulation boxes were built to contain 35,621 (39,508) atoms together with three (three) sodium ions and 10,952 (12,241) TIP3P waters [14] for glutamine-free (-bound) GBP. The MD simulations for both systems were carried out in the same manner as that for ADK.

#### ABC transporter

The crystal structure of P-glycoprotein homolog, *Cyanidioschyzon merolae* CmABCB1, without a ligand molecule was used as the initial structure for the MD simulations of the multidrug ABC transporter (PDB: 3wmg_A) [21]. The missing loops of the structure were modeled and the mutations at residues 277–279 (VVV) were replaced by wild type amino acids (GAA) using MODELLER [22]. The simulation system was constructed as follows: the protein was embedded in an equilibrated lipid bilayer membrane of palmitoyl-oleoyl-phosphatidylcholine (POPC) with the transmembrane region estimated by TMHMM [23]. The simulation box of the drug-free state contained 249,057 atoms with 60,023 TIP3P waters [14] and 192 sodium chloride ions to make the experimental ionic concentration (= 150 mM) [21]. Rhodamine 6G was used as a drug molecule of the bound state to be transported. The possible initial positions of rhodamine 6G were modelled with the simulation software GOLD [24], finding one cavity for each subunit. To construct the drug-bound model, a single drug molecule was placed at the cavity of one subunit in the drug-free structure (see Results section for details). The model containing a single drug molecule in a subunit was used in the drug-bound simulation because it was found in a test simulation that two drugs positioned in the two subunits tended to immobilize the inward-open structure much more strongly than in the drug-free state.

The simulations were performed with the MD program MARBLE [25] with the NPT condition, wherein the *x* and *y* axes defining the membrane in-plane were isotropically scaled. The CHARMM 36 all-atom parameter [26] was used for the potential energy function. The force field of rhodamine 6G was modeled as in refs [27–29]. Electrostatic interactions were calculated using the particle-mesh Ewald method [15]. The cut-off length of the Lennard-Jones potential was 10 Å. The symplectic integrator for rigid bodies was used to constrain the bond lengths and angles involving hydrogen atoms [25], allowing the time step to be 2.0 fs. The Motion Tree was built using only 850 C_α_ atoms within the secondary structural elements to better clarify the structural dynamics of the huge protein.

## Results

### Motion Tree of ADK dynamics

**Fig 1:**
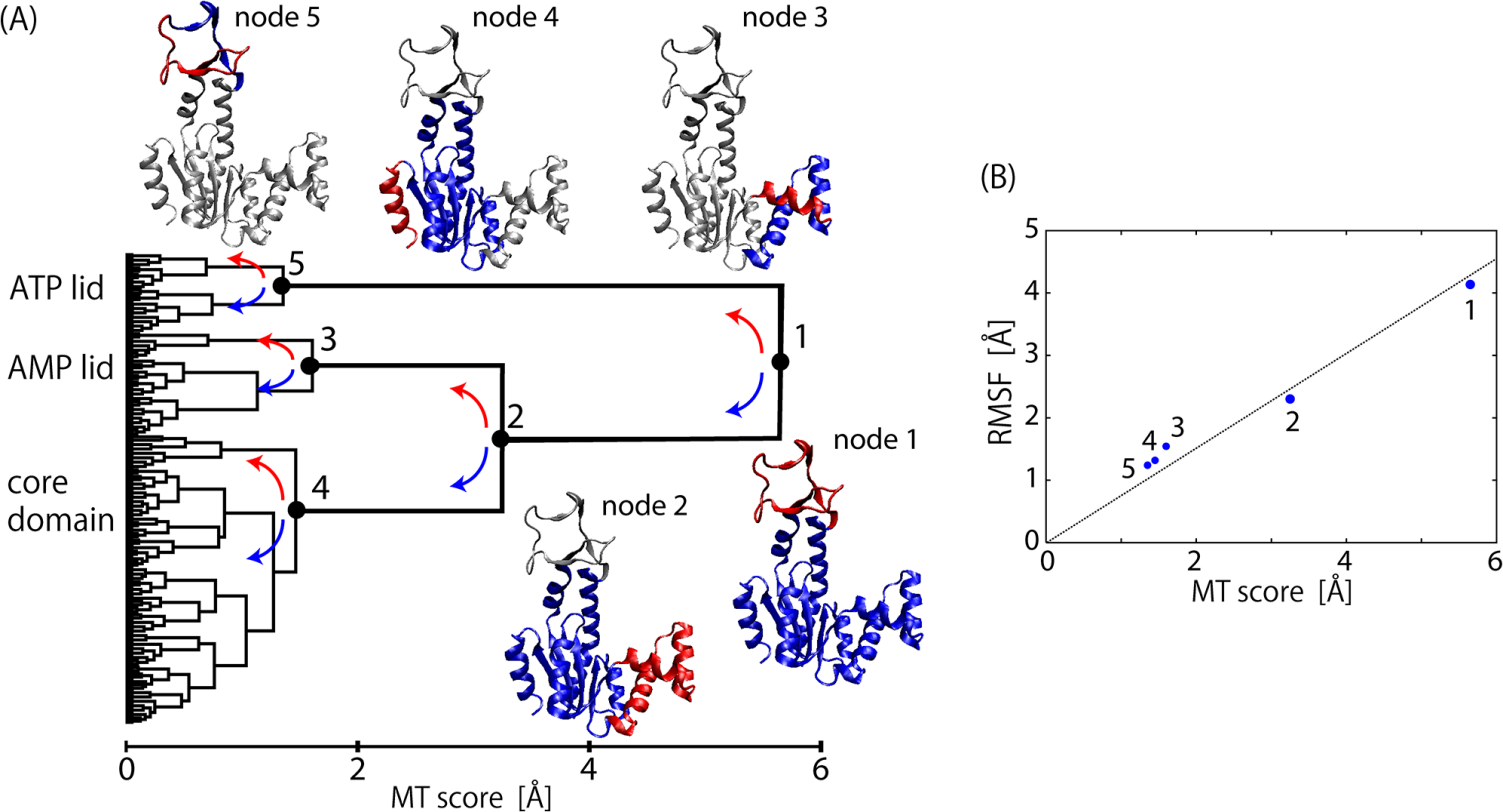
Motion tree for substrate-free ADK. (A) Motion Tree constructed from 50-ns dynamics of substrate-free adenylate kinase. Five nodes are shown with corresponding parts of ADK structure in blue (larger domain) and red (smaller domain). (B) RMSF value for smaller (red) domain after fitting to corresponding larger domain is plotted at each node as a function of MT score. Dotted line is least square fit with zero at origin.

Fig 1A shows an illustrative example of a Motion Tree constructed from the 50-ns MD simulation of adenylate kinase (ADK) in the ligand-free state. ADK has two lids to bind ATP and AMP, which undergo structural changes to the closed form on ligand binding. The Motion Tree is interpreted by going down from the root to the leaves as follows: The root denotes the largest cluster composed of all residues in ADK, and is divided at node 1 into two sub-clusters, the first corresponding to the ATP-lid (residue 123–158) and the second composed of the AMP-lid and the core domain. The height from the bottom of the tree (MT score at node 1; *s*_1_) is the largest (*s*_1_ = 5.7 Å), indicating that the fluctuation between the ATP-lid and the AMP-lid/core domain is the largest in ADK dynamics, and that the fluctuations occurring within the two sub-clusters are smaller than *s*_1_. Node 1 describes the inter-domain motion between the ATP-lid and the AMP-lid/core domain in terms of protein dynamics. However, these domains are not completely rigid bodies but contain intra-domain motions, which are represented by descendant nodes in the tree. Node 2 thus exhibits the largest motion within the AMP-lid/core domain. The intra-domain motion at node 2 can be interpreted as inter-domain motion between the AMP-lid and the core domain since the node separates the AMP-lid/core domain further into the AMP-lid (residue 31–77) and the core domain. The MT score *s*_2_ (= 3.3 Å) is smaller than *s*_1_, i.e., the fluctuation between the AMP-lid and the core domain is smaller in amplitude than that between the ATP-lid and the AMP-lid/core domain. Nodes 3 to 5 denote the intra-domain fluctuations in the three domains, the ATP-lid, AMP-lid, and the core domain, which are further divided into sub-clusters. Their MT scores are less than half of *s*_1_ and *s*_2_ (*s*_3_ = 1.6, *s*_4_ = 1.5 and *s*_5_ = 1.4 Å) and indicate the dominance of the two largest inter-domain motions in ADK dynamics.

These descriptions of ADK dynamics are in good agreement with those reported in the literatures [30–33]. Moreover, the Motion Tree of ligand-free ADK dynamics in Fig 1A is very similar to that from two static structures, i.e., the average structure of the present simulation and the crystal structure of the bound state (PDB: 2eck) [34] (S1 Fig). This clearly indicates that the structural change on ligand binding from the open to the closed structure can be regarded as a process of linear response, where structural change is mostly determined by fluctuations in the ligand-free state [35].

In summary, the advantage of the Motion Tree is the hierarchical description of protein dynamics, which systematically illustrates a clear separation into various sizes of rigid domains and associated inter-domain motions ranging from overall collective motions to local vibrations. This is in contrast to methods solving eigenvalue problems that focus on large domain motions like PCA, which may obscure the detection of local but functionally-relevant motions. Although the MT score is based on inter-domain distance fluctuations, there is a close correlation between the MT score and the root mean square fluctuation (RMSF) of associated inter-domain motion as shown in Fig 1B. Therefore, the MT score at a node can be regarded as a good measure for motional amplitude between the two domains separated at the node. This feature is in contrast to non-hierarchical clustering-methods [6–10] which focus only on the domain identification and yield no information on the amplitudes and interrelationships among motions of the rigid bodies.

### Motion Trees for GBP dynamics with and without bound glutamine

As described above, the Motion Tree provides a simplified and intuitive representation of protein dynamics inherent in an MD simulation trajectory. Therefore, a number of Motion Trees are also useful to compare protein dynamics simulated under different conditions, such as ligand/substrate binding. Here, we compared two Motion Trees calculated from the 50-ns MD trajectories of ligand-free and ligand-bound forms of glutamine binding protein (GBP) to find changes in the dynamics of GBP upon glutamine binding.

**Fig 2:**
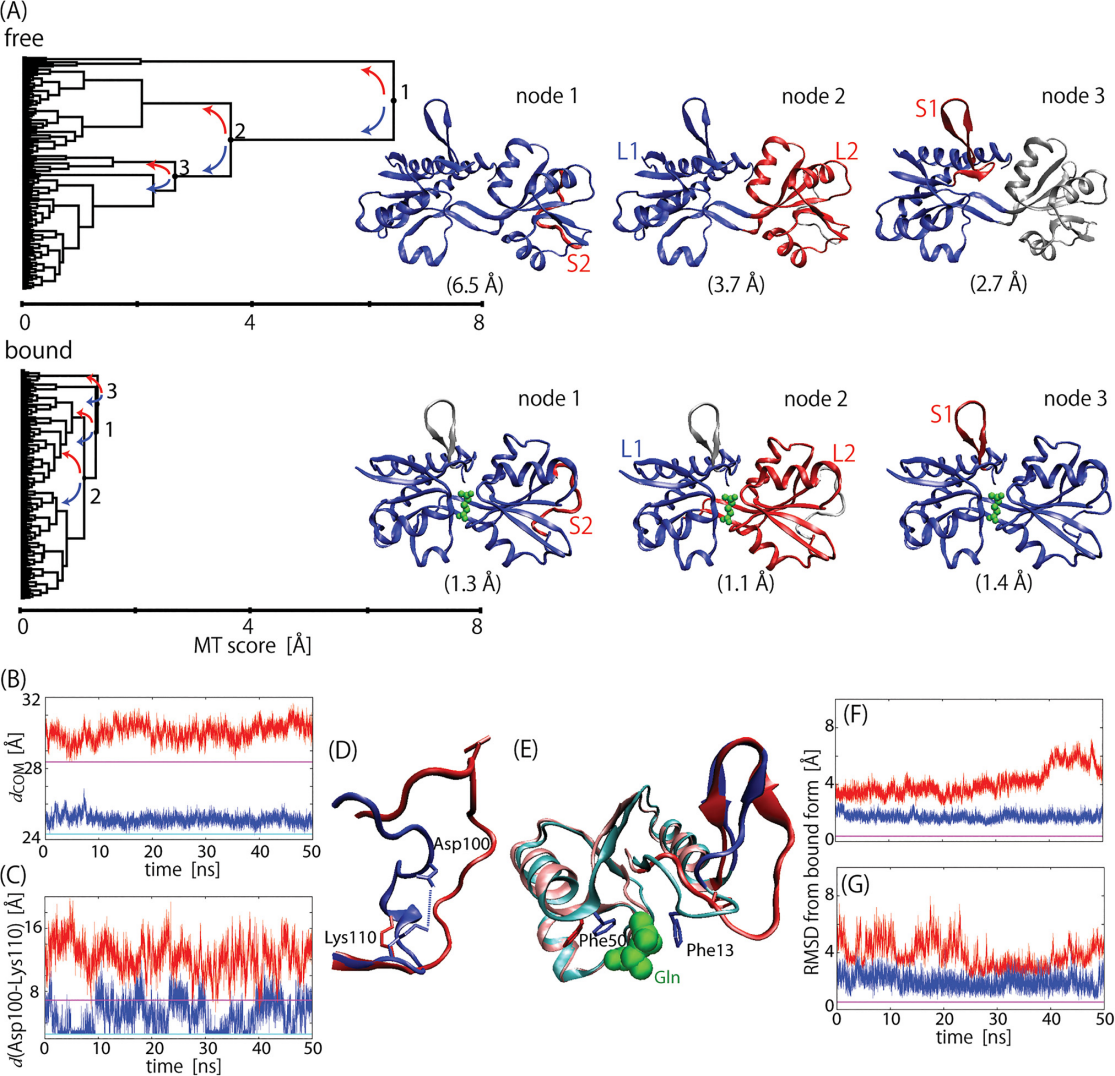
Motion trees for ligand-free and ligand-bound GBP. (A) Motion Trees constructed from 50-ns trajectories of ligand-free and ligand-bound GBP, where flexible C-terminuses (residues 222–226) were ignored. Three nodes are shown with corresponding parts of GBP structures in blue (larger domain) and red (smaller domain). Node numbers for ligand-bound form are given so that they have same structural assignments as those for ligand-free form. MT score at each node is given in parenthesis. Four moving elements identified are two domains, L1 (residue 5–10, 28–89, 183–224 (free) and 5–16, 27–82, 187–224 (bound)) and L2 (90–97, 108–182 (free) and 83–95, 106–186 (bound)), and two loops, S1 (11–27 (free) and 17–26 (bound)) and S2 (98–107 (free) and 96–105 (bound)). (B) Center-of-mass distances between L1 and L2, and (C) distances between nearest polar atoms belonging to Asp100 and Lys 110 in S2. Red plots are for free states and blue plots are for bound states. Values in crystal structures are also shown for free form (magenta) and bound form (cyan). (D) Simulated structures of S2 at 50 ns for free (red) and bound (blue) forms. Ion pair between side chains of Asp100 and Lys 110 is indicated by dotted line. (E) Simulated structures of S1 at 50 ns for free (red) and bound (blue) forms. The structures near S1 loop are also indicated by pink (free) and cyan (bound), as well as bound glutamine and side chains of Phe 13 and 50. (F) and (G) show RMSD values of residues 11–16 and 17–26 after fitting L1 to that of ligand-bound form of the crystal structure. Color scheme is same as that in (B) and (C).

Fig 2A shows the Motion Trees derived, each of which successfully provides a complete picture depicting both large domain motions and local motions in a single tree diagram. These trees indicate that two large domains, L1 and L2, and two small loops, S1 and S2, contributes significantly to the GBP dynamics, although their MT scores, or the heights of the trees, greatly differ from each other, indicating changes in domain fluctuations on ligand binding. The comparison demonstrates that ligand binding reduces the amplitude of domain motions between L1 and L2 by more than three times ([*s*_2_ of free]/[*s*_2_ of bound] = 3.7 Å/1.1 Å). Dynamical stiffening seen in the Motion Trees is consistent with the MD trajectories. Fig 2B shows large difference in the distance (*d*_COM_) fluctuation between the centers-of-mass of L1 and L2. Reduction in the amplitude can also be seen in the loop motions. A five times decrease can be found in the S2 loop ([*s*_1_ of free]/[*s*_1_ of bound] = 6.5 Å/1.3 Å). However, this change in dynamics was found to be mostly due to the side-chain polar contact between Asp100 and Lys 110; the bound state had contact for about half the simulation time, whereas this was broken to largely fluctuate S2 in the free form (Fig 2C and 2D). This change simply originated from the difference between the two crystal packing structures used in the MD simulations as the initial structures (Panel A in S2 Fig), and was probably irrelevant to ligand binding.

The motion of the S1 loop (node 3) captured in the Motion Tree was much more significant as this loop was definitely affected by ligand binding. Phe13 in the bound form recognized the glutamine molecule with the hydrophobic sandwich with Phe50 (Fig 2E). Such aromatic stacking of the ligand is also found in other periplasmic binding proteins such as lysine-arginine-ornithine binding protein and histidine binding protein, which may help orient the ligand into a favorable conformation in the initial stage of ligand binding [19]. Ligand binding shortens S1 by six residues from residues 11–26 to 17–26. The first six residues 11–16 belong to L1 in the bound state. The ligand molecule bridges Phe50 in L1 to Phe13 and reduces the flexibility of residues 11–16 relative to L1 (Fig 2F). However, even without direct interaction with ligands, the motions of residues 17–26 appear to be constrained by ligand binding ([*s*_3_ of free]/[*s*_3_ of bound] = 2.7 Å/1.4 Å and Fig 2G). Note that the crystal structures of ligand-free and-bound forms do not show any significant motion in S1, as seen in the Motion Tree comparing the crystal structures (Panel B in S2 Fig), indicating that S1 did not change the conformation in ligand binding in the crystal environment, unlike the S1 motions observed in the solution environment of the MD simulations.

### Dynamic change in inward-open ABC transporter in binding of rhodamine 6G

**Fig 3:**
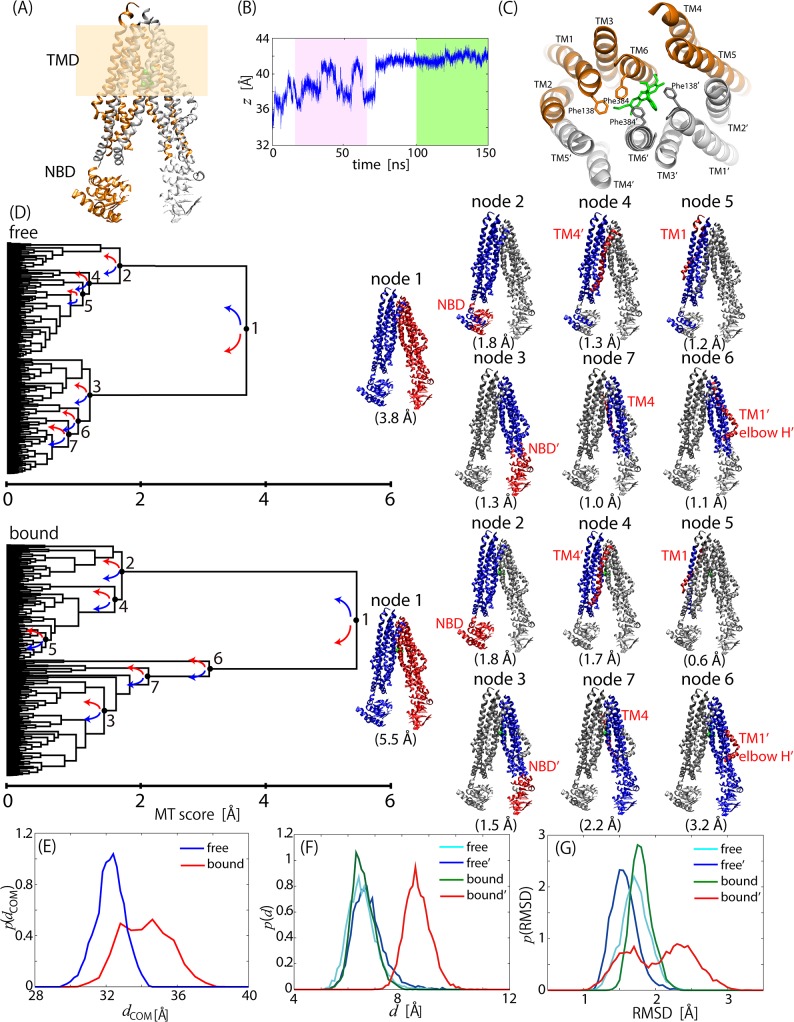
Motion Trees for drug-free and-bound CmABCB1. (A) Structure of CmABCB1. Two dimer chains are in orange and gray. Each of right and left subunits consists of TMD and NBD, where membrane spanning regions are colored in light brown. (B) Migration of rhodamine 6G center-of-mass along *z*-axis in 150-ns drug-bound simulation. Green and pink boxes correspond to simulation time ranges used for calculating Motion Trees in Fig 3D and Panel B in S3 Fig (C) Stable binding site including rhodamine 6G (cyan) and two phenylalanine side-chains of 138' and 384'. Colors of two dimer chains are same as those in (A). (D) Motion Trees calculated from last 50-ns trajectory of drug-free and-bound states. Nodes and corresponding structures are indicated with same colors, blue for larger and red for smaller portions. (E) Probability distribution of distance between center-of-masses of two subunits for drug-free (blue) and drug-bound (red) states. (F) Probability distribution of distance between center-of-masses of aromatic rings of Phe138 and Phe384 for left (cyan) and right (blue) subunits of the drug-free state, and for left (green) and right (red) subunits of the drug-bound state. (G) Probability distribution of RMSD for elbow-helix/TM1 relative to other TMD region from the crystal structure of the free form. Color scheme is same as that in (F).

The ATP-binding cassette (ABC) multidrug transporter is an ATP-dependent efflux pump with a broad range of drug specificity. Drug transport occurs during the process of structural transformation from inward-open to outward-open structures driven by ATP binding and hydrolysis. Here, we chose a eukaryotic member of the ABC multidrug transporter family, CmABCB1, as the simulation system. CmABCB1 adopts a home-dimeric architecture with each subunit consisting of a nucleotide binding domain (NBD) that binds and hydrolyzes ATP to power the transport process and a transmembrane domain (TMD) that creates the translocation pathways for substrates (Fig 3A). Each TMD is composed of an N-terminal elbow helix (elbow H) followed by six transmembrane helices (TM1-6) and two short intracellular helices, of which TM4 and TM5 are swapped between the two TMDs, that is, six transmembrane helices of a TMD are TM1, TM2, TM3, TM4', TM5', TM6 (left TMD in Fig 3A), and the other are TM1', TM2', TM3', TM4, TM5 and TM6' (right TMD in Fig 3A), where “'” indicates the helix of another chain.

It has recently been shown that the ATPase activity in CmABCB1 is activated by binding of drug molecules such as rhodamine 6G [21], that is, drug binding enhances the ATPase activity and thus promotes structural transformation. We compared the Motion Trees derived from two MD simulations of an inward-open structure with and without rhodamine 6G to examine how drug binding influenced protein dynamics. The drug-bound model was constructed by placing a single drug molecule at the cavity of the right TMD in the drug-free structure (Panel A in S3 Fig). It was observed in the 150-ns bound-state MD simulation that the drug molecule migrated by ~7 Å in the right TMD from the initial position between Ile354 (TM5) and Met 391'(TM6') toward the extracellular side and reached a stable position between Phe138' (TM1') and Phe384' (TM6') after ~80 ns (Fig 3B and 3C, and Panel A in S3 Fig). Thus, the last 50-ns trajectory was used to calculate the Motion Tree for the dynamics of the drug-bound state.

The Motion Trees in Fig 3D demonstrated that node 1 divided a molecule into left and right subunits, which have the largest fluctuation (*s*_1_ = 3.8 Å for the free state and 5.5 Å for the bound state). Drug binding increased the *s*_1_ value. The increase in the inter-subunit fluctuation can also been seen in the increased inter-subunit fluctuations together with a separation of the inter-subunit distance (Fig 3E). The increased fluctuation and the opening motion of the two subunits occurred already at the initial stage of the simulation before the drug molecule moved to the extracellular side, as is shown by the similar MT score (*s*_1_ = 5.4 Å) for the trajectory between 20 to 70 ns (Panel B in S3 Fig). This suggests that the drug insertion enhanced the inter-subunit motions to promote the transport of the drug molecule to the extracellular region. Three moving parts were also identified relative to the core region in the descendant node; NBDs, elbow-helix/TM1s, and TM4s (or parts of them; see the Motion Trees and structures in Fig 3D) as the most flexible parts within each subunit (see nodes 2–7 in Fig 3D). The comparison of the two Motion Trees indicated that the drug binding had no significant influence on the fluctuations of NBDs and TM4', but that TM4 exhibited the increase in MT score, [*s*_7_ of bound] / [*s*_7_ of free] = 2.2 Å / 1.0 Å. A further analysis of the RMSD distribution of TM4 from the drug-free crystal structure indicated that broadening upon drug binding appeared on both sides of the distribution (Panel C in S3 Fig). Therefore, it is considered that drug binding randomly enhanced the fluctuation of TM4 as well as the amplification of the inter-subunit motions seen at node 1 (see Fig 3D). TM4 is actually attributed to the unstructured loop at the back-mutation sites (residues 277–279 for TM4; GAA/VVV), which is considered to play the role of a gatekeeper for the drug entrance gate [21].

A remarkable difference between the Motion Trees for the drug-free and-bound states is seen at elbow-helix/TM1; for the drug-bound state, the fluctuation in elbow-helix'/TM1' in the right TMD (*s*_6_ = 3.2 Å) is much larger than the value in the left TMD (*s*_5_ = 0.6 Å), while for the drug-free state the fluctuations in elbow-helix/TM1s in both TMDs are small and comparable (*s*_6_ = 1.2 and *s*_5_ = 1.1 Å). This significant increase in fluctuation in elbow-helix'/TM1' is due to the intervention of the drug molecule between Phe138' (TM1') and Phe 384' (TM6') found in the stable binding pose (see Fig 3C), which is strengthened by the result that the corresponding MT score is much smaller than that in the MD trajectory before drug intervention (*s*_6_ = 0.9 Å, see Panel B in S3 Fig). Note that TM1s and TM6s have tight contact in the inward-open structure, while they are largely separated in the outward-open structure [21]. Therefore, drug intervention into the two helices is considered to be a trigger for the structural transformation. Kinetic analysis of ATPase activity actually indicated that alanine mutation of the two Phe residues exhibited reduced affinity to rhodamine 6G [21]. It can clearly be seen in Fig 3F that drug intervention increases the distance between the two Phe side chains by about 2 Å. Consequently, the elbow-helix'/TM1' of the drug-bound subunit started to fluctuate much more than those of the other portions (Fig 3G).

In summary, the binding of rhodamine 6G led to the change in the dynamics of the transporter in the following two stages. First, when with the drug molecule was inserted at the initial position, the two subunits underwent the opening motion and increased their fluctuations. The loosed interaction and increased fluctuation between the subunits allowed the drug molecule to move to the extracellular side. Finally, the dissociation between Phe138' (TM1') and Phe 384' (TM6') via the drug intervention occurred the subsequent increase of local motion in elbow-helix'/TM1', which would have been a trigger for the large structural rearrangement of CmABCB1. Both of a large-scale subunit motions and a tiny change in the dynamics of elbow-helix'/TM1' hidden behind the large subunit motions were successfully revealed by analyzing the complicated MD trajectories of the huge protein complex via Motion Trees.

## Discussion

Protein functional motions inherently recorded in MD simulation trajectories are obscured by dynamical complexity with a wide range of amplitudes and timescales ranging from small localized vibrations to large collective motions. According to the recent increase in simulation timescales and system sizes of target proteins, it has become more important to capture a straightforward and intuitive picture of protein dynamics from MD trajectories. To this end, a novel description of protein dynamics is proposed based on the hierarchical clustering of fluctuations in inter-atomic distances calculated from an MD trajectory. The tree diagram thus constructed, named a “*Motion Tree*”, describes the “multi-scale” picture of protein dynamics including all sizes, magnitudes, and cooperativity of rigid-body-like motions hierarchically along with the amplitudes of inter-domain fluctuations. The comparison in the Motion Trees of complex protein dynamics simulated under different conditions, such as ligand/substrate binding, has provided an at-a-glance view of the changes in both collective and local motions related to the associated protein functions.

The three main advantages of Motion Tree in the analysis of complex MD trajectories are as follows: First, hierarchical clustering of atom-atom distance deviations leads directly to a description of protein dynamics via a set of hierarchically defined inter-domain motions together with the associated amplitudes, which is in contrast to non-hierarchical clustering methods [6–10]. The hierarchical clustering also allows both collective and local motions to be sensitively identified. Local motions, which are sometimes closely related to protein functions, are usually hidden behind the complicated MD trajectory of large degrees of freedom and difficult to be detected with the conventional methods such as PCA. Second, the calculation of Motion Tree does not require any prior knowledge other than the distance deviation matrix. Therefore, Motion Tree can be constructed immediately after the MD simulation is completed. The dynamics overview found in Motion Tree will be useful as a first step for subsequent analyses of the MD trajectory. Third is methodological flexibility. The matrix for clustering, **D**, can be calculated on the arbitrary description level of protein molecules, such as the residue-level using C_α_ atoms/the detailed all-atom level and the whole molecule/selected parts of the molecule. The definition of the metric for hierarchical clustering is also arbitrary. It is possible to use higher-order distance fluctuations for **D** to include the influence of anharmonic motions, such as diffusive structural changes or “rare events” that appeared in MD simulation trajectories.

The program code is available from the authors on request.

## Supporting Information

S1 Fig Motion Tree calculated using Eq 1 from two structures, average structure of 50-ns substrate-free ADK simulation and crystal structure of substrate-bound ADK (PDB: 2eck). Node numbers are given so that they have same structural assignments as those in Fig 1A.
(TIF)

S2 Fig (A) Crystal structures of S2 for free (pink) and bound (cyan) forms. Ion pair between side chains of Asp100 and Lys 110 is indicated by dotted line. (B) Motion Tree calculated using Eq 1 from two crystal structures of ligand-free and-bound GBP. Node numbers are given so that they have same structural assignments as those for ligand-free form (top of Fig 2A).
(TIF)

S3 Fig (A) The initial and final rhodamine 6G structures in the MD simulation are indicated in orange and magenta. The side-chains of the relevant amino acids are indicated. See Fig 3B for details. (B) Motion Trees calculated from drug-bound CmABCB1 trajectory from 20 to 70 ns (pink box in Fig 3B). Nodes and corresponding structures are same as those in Fig 3D. (C) Probability distribution of RMSD for TM4 relative to other TMD region from the crystal structure of the free form. TM4 (cyan) and TM4' (blue) of the drug-free state, and TM4 (green) and TM4' (red) of the drug-bound state.
(TIF)
