# Supplementary figures and images for: Motion Tree Delineates Hierarchical Structure of Protein Dynamics Observed in Molecular Dynamics Simulation
Source: PLoS One. 2015 Jul 6;10(7):e0131583. doi: 10.1371/journal.pone.0131583 (PMC4492737; doi:10.1371/journal.pone.0131583)

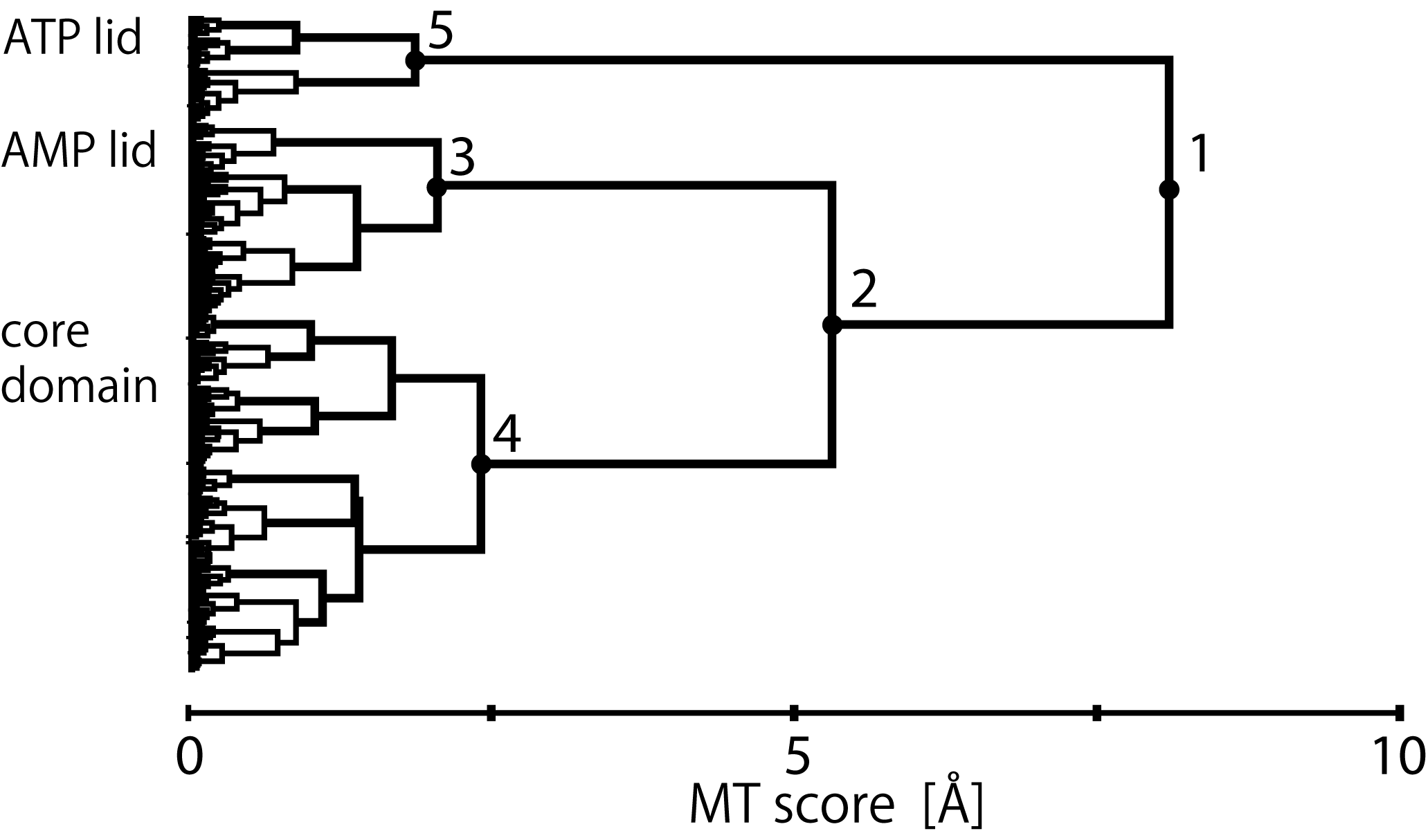

Supplement: S1 Fig — Node numbers are given so that they have same structural assignments as those in Fig 1A. (TIF) [file pone.0131583.s001.tif]

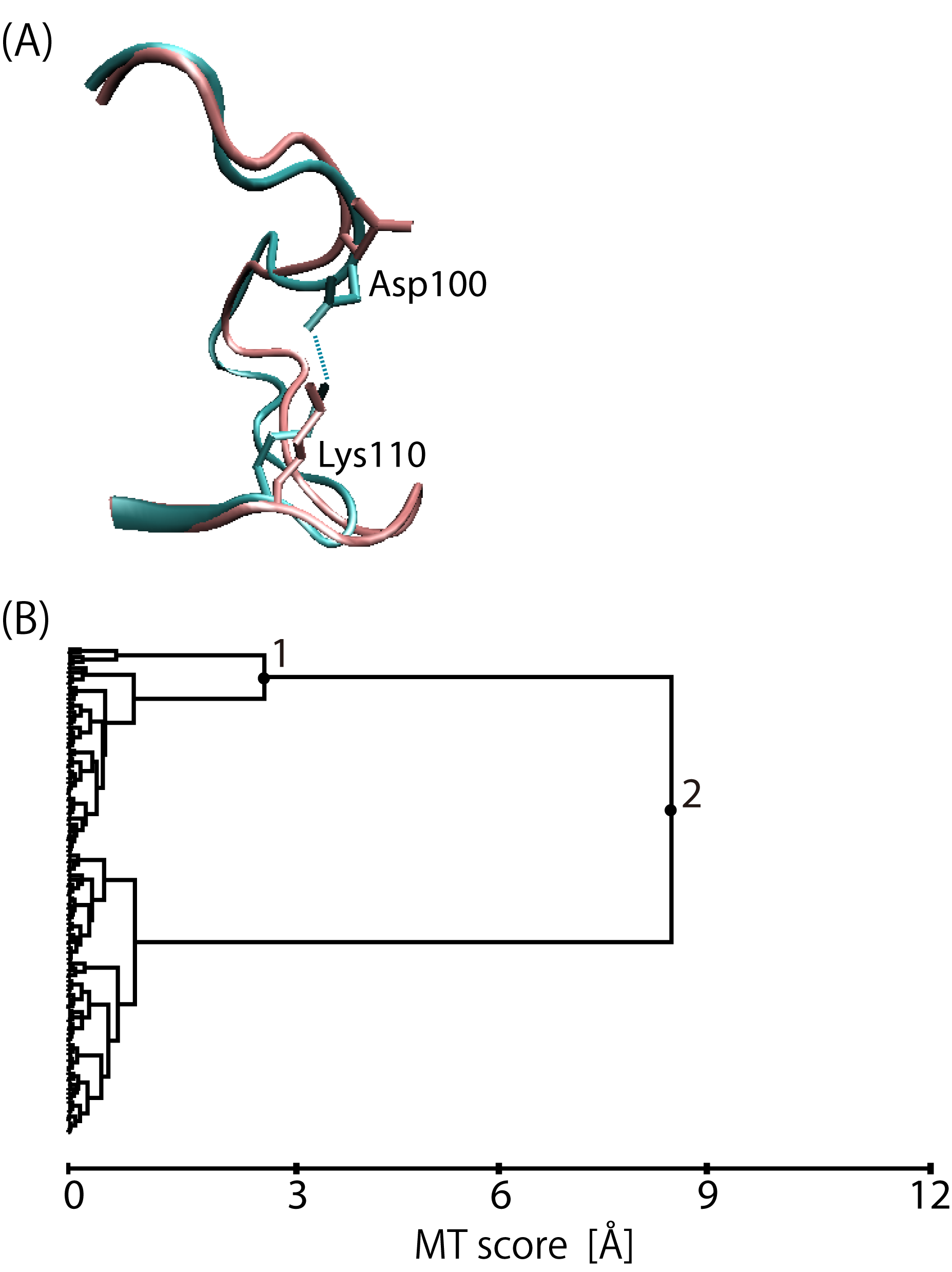

Supplement: S2 Fig — (A) Crystal structures of S2 for free (pink) and bound (cyan) forms. Ion pair between side chains of Asp100 and Lys 110 is indicated by dotted line. (B) Motion Tree calculated using Eq 1 from two crystal structures of ligand-free and-bound GBP. Node numbers are given so that they have same structural assignments as those for ligand-free form (top of Fig 2A). (TIF) [file pone.0131583.s002.tif]

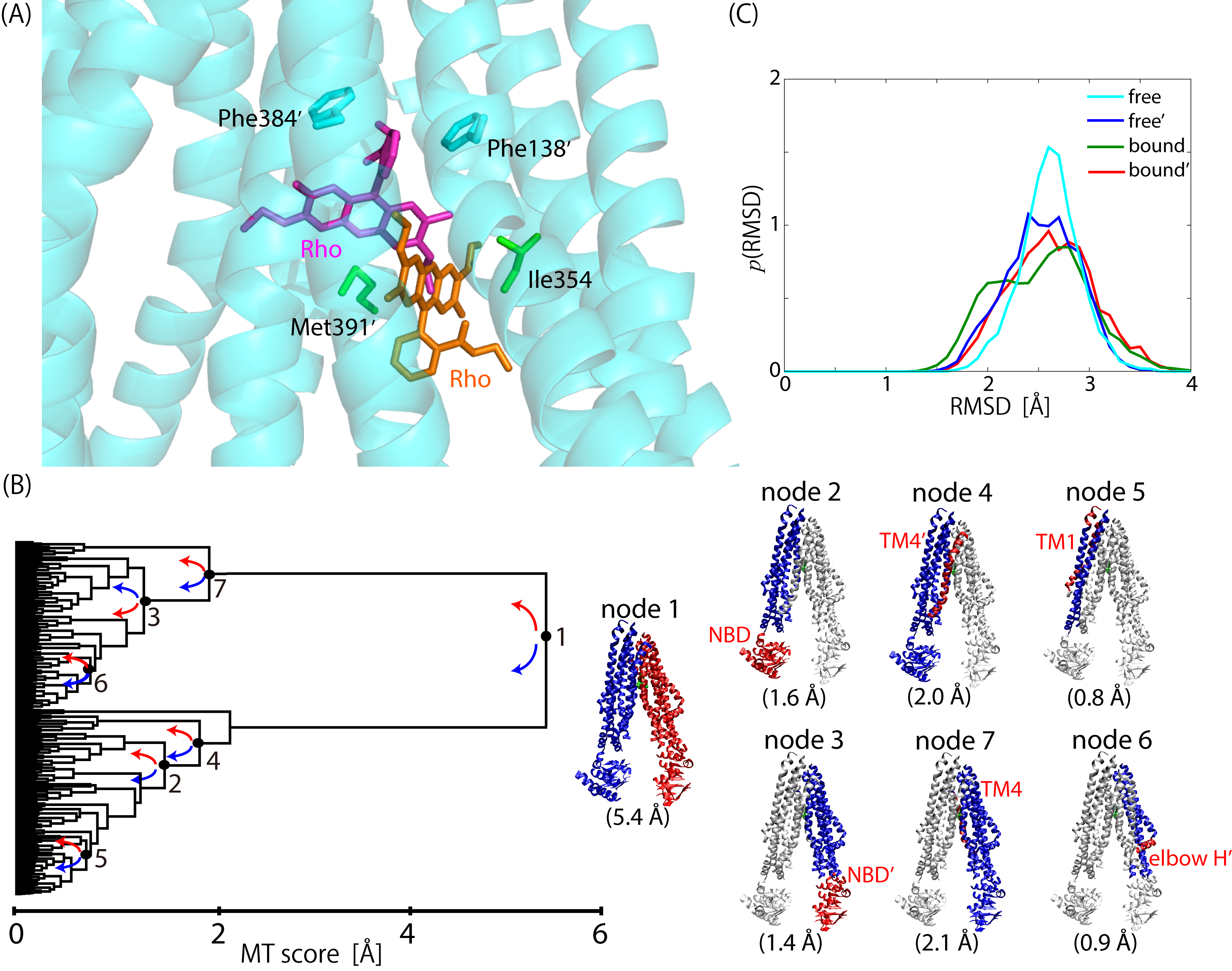

Supplement: S3 Fig — (A) The initial and final rhodamine 6G structures in the MD simulation are indicated in orange and magenta. The side-chains of the relevant amino acids are indicated. See Fig 3B for details. (B) Motion Trees calculated from drug-bound CmABCB1 trajectory from 20 to 70 ns (pink box in Fig 3B). Nodes and corresponding structures are same as those in Fig 3D. (C) Probability distribution of RMSD for TM4 relative to other TMD region from the crystal structure of the free form. TM4 (cyan) and TM4' (blue) of the drug-free state, and TM4 (green) and TM4' (red) of the drug-bound state. (TIF) [file pone.0131583.s003.tif]
